# Effect of carbon anode production parameters on anode cracking

**DOI:** 10.1007/s42452-020-04133-8

**Published:** 2021-01-25

**Authors:** Salah Amrani, Duygu Kocaefe, Yasar Kocaefe, Dipankar Bhattacharyay, Mohamed Bouazara, Jules Côté

**Affiliations:** 1 Alouette Research Chair On Carbon, Aluminium Research Centre (REGAL), UQAC/Aluminerie University of Québec at ChicoutimiUniversity of Québec, Chicoutimi, Québec G7H 2B1 Canada; 2 Aluminerie Alouette Inc, 400, Chemin de la Pointe-Noire, Sept-Îles, Québec G4R 5M9 Canada; 3 Centurion University of Technology and Management, R.Sitapur, Odisha 752050 India

**Keywords:** Aluminum production, Carbon anode, Vibro-compaction, Anode baking, Characterization

## Abstract

Carbon anodes are used in the electrolytic production of aluminum. The quality of anodes is directly related to the production cost, carbon and energy consumption, and environmental emissions. It is desired that the anodes have high density, low porosity/cracks, low electrical resistivity as well as low air and CO_2_ reactivities. Low resistivity of anodes reduces energy required to produce aluminum during electrolysis. The presence of cracks and pores increases the anode electrical resistivity. Therefore, it is important to know how and when the pores and cracks form during the anode production so that the necessary actions could be taken to prevent their formation. A study was carried out to investigate the effect of different anode production parameters such as anode composition, type of raw material used, time and top-former bellow pressure of vibro-compactor, green anode cooling medium, and heating rate used during baking on the crack formation. The anodes are fabricated at the carbon laboratory of University of Quebec at Chicoutimi (UQAC) and characterized by measuring their properties (density, electrical resistivity, and surface crack density). The anode properties, hence the anode quality, were correlated with the anode production parameters. Also, their tomographic analysis was carried out to visualize and quantify the internal cracks.

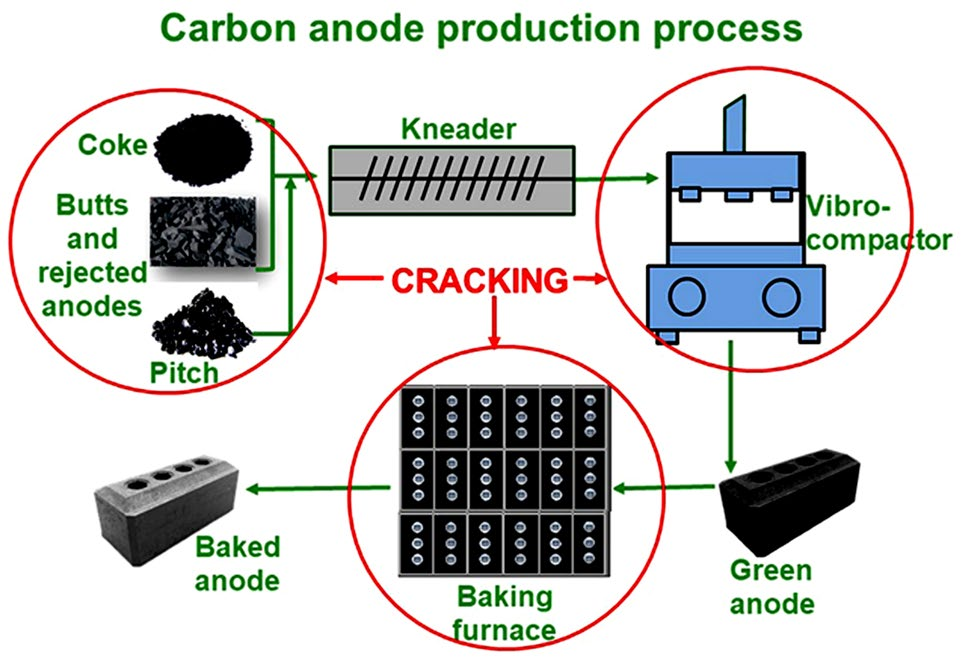

## Introduction

Aluminum is produced by electrolysis using the Hall-Héroult process according to the reaction:

1 $$ 2{\text{Al}}_{2} {\text{O}}_{3} + 3{\text{C}} \to 4{\text{Al}} + 3{\text{CO}}_{2} $$

The carbon required for aluminum production via electrolytic alumina (Al_2_O_3_) reduction is supplied by the carbon anodes which are consumed during electrolysis. The cost of anodes is around 15% of the total cost of aluminum production [1]. However, this can increase to 25% due to over-consumption of carbon if the anode quality is poor. The quality of the anodes has a major influence on the efficiency of the electrolysis. Each anode has a certain porosity and some cracks, which is inevitable. However, the presence of excessive cracks/pores is considered a sign of poor anode quality since they increase the electrical resistivity of anodes, hence the energy needed for electrolysis. They also decrease anode density. In addition, the utilization of poor-quality anodes results in shorter anode life (increased anode consumption and production cost) and increases greenhouse gas (GHG) emissions.

Modern electrolysis cells use prebaked anodes. During anode production, dry aggregate (petroleum coke, rejected green and baked anodes, and butts) with a desired granulometry is mixed with coal tar pitch, which acts as the binder, to produce anode paste. Then, anode paste is compacted in a vibro-compactor or a press to obtain green anodes, which are baked in large furnaces to produce baked anodes. These are used in electrolysis after rodding. All the anode production parameters affect the anode quality.

The current study was carried out to investigate the effect of the different parameters (anode composition, raw materials, time and top-former bellow pressure of vibro-compactor, medium used to cool the green anodes, and heating rate used during baking) to determine the influence of production conditions on anode cracking.

There are some studies reported in the literature on the formation of cracks and their effect on anode quality. The quality of raw materials and the anode recipe used can affect the anode quality. Utilization of suitable anode recipe, where fines are filling the spaces between the particles are important. Use of high quantity of butts might result in cracks since the thermal expansions of butts and the rest of the dry aggregate are different. The stress created during baking at the positions where butt is present might cause new cracks to form [2, 3].

Utilization of coke with high expansion coefficient and low grain stability can form cracks [4]. Coke with high resiliency can also cause cracking due to spring-back effect. High sulfur content cokes may lead to cracking if the baking temperature is high. Suitable levels of pitch content and aggregate sizing are required to prevent any excessive shrinkage or expansion, thus, crack and porosity formation [3]. If the anode is under-pitched (not enough pitch), air is trapped between the aggregate particles as well as in the pores of the particles. These anodes are consumed quickly in the cell. If the anode is over-pitched (too much pitch), the cracks can form due to high quantity of volatile release during baking [5]. In addition, butts contain sodium which increases the anode reactivity, thus anode consumption [6].

Binder pitch can influence the green anode density of the anodes. Good and homogeneous pitch distribution can reduce the air trapped between the coke aggregates in green anodes [7]. Different plants use different particle size distribution (anode recipe) to have good quality anodes. Particle size distribution not only affects the anode density, but it also influences the air permeability [8], pore size distribution [9], mechanical strength, electrical resistivity [3], reactivity and even chemical composition of anodes [10].

Mixing parameters (mixing time, mixing speed, mixing temperature) are important [3, 11] for anode quality. If the mixing of dry aggregate and pitch is not done properly, the anodes become non-homogeneous. Then, the cracks will form in pitch rich regions during pitch devolatilization. Homogeneous anode paste reduces the pitch consumption and high local volatile release leading to lower internal pressure and cracking rate during anode baking [12, 13].

Vibro-compaction parameters (paste temperature, forming temperature, and vibration time) have to be well adjusted to avoid excessive cracking [4]. If the compaction time is too short, the anode is under-compacted, which decreases the anode density and increases the porosity as well as dusting. On the other hand, long compaction times lead to over-compaction of the anode. This results in stress formation yielding to cracks and delamination [15]. Hulse [3], Tkac [9], and Sanogo [16] showed the effect of compaction time on the anode properties and proposed a method to determine the optimum compaction time**.** The results indicated that there is indeed an optimal vibration time. Exceeding this time deteriorates the mechanical properties and increases the electrical resistivity. In addition to compaction time, compaction force (load and vibro-compactor top-former bellow pressure), vibration frequency, and the utilization of vacuum are the parameters which can affect the anode quality [14]. Furthermore, the utilization of vacuum during vibro-compaction helps remove the trapped air between the particles as well as in the coke pores and facilitates the penetration of pitch.

The green anodes are baked for about two to three weeks in baking furnaces at temperatures up to around 1150 °C before being used in electrolysis. Pitch carbonizes and links aggregate particles together [17]. During baking, there are two temperature regions that are critical for cracking. Up to 150 °C, stresses formed during compaction and paste cooling are released leading to anode expansion [18, 19]. If these stresses exceed a certain limit, cracks can form. Cracks can appear as well during baking in the temperature range of 200 °C to 600 °C if the gas pressure builds up excessively due to devolatilization of pitch especially if high heating rates are used [4, 20, 24]. As previously mentioned, a better distribution of pitch in the anode block causes a reduction in the internal pressure (due to the devolatilization) during baking; this leads to an anode with fewer cracks [13]. The anode life and carbon consumption is strongly affected by baking parameters [21]. The cracking can also be provoked if large temperature gradients exists in the anode [22]. Sendid and Courau [23] reported that the propagation of existing cracks is also a major source of anode cracking.

This paper reports the effect of different operational parameters on the cracking of anodes produced, consequently on anode properties. The anodes were produced using the same raw material and under the same operating conditions except the parameter to be studied is varied. Also, tomographic analyses of the anodes were carried out. This approach makes the systemic investigation of cracking possible. Anodes were characterized measuring the apparent density, electrical resistivity, surface crack density, internal defects (cracks/pores), and the optical density. The number and percentage of internal defects were calculated from the tomography results with the software developed by the carbon group at UQAC in order to visualize and quantify the cracks/pores.

Previously, the carbon group of UQAC developed a special equipment (SERMA) to determine how the anode electrical resistance varies with time during baking [24, 25]. The resistivity variation is directly proportional to crack formation since cracks increase resistivity. This study shows the evolution of crack formation during baking. Many parameters affect the crack formation as given in the present article, and the cracks form primarily during baking. It should be noted that it is impossible to determine exactly which crack forms at what time in such materials. Materials and methods used are described in the following section. Then, the results are presented and discussed. At the end, the conclusions are given.

## Materials and methods

### Properties of raw materials

Three petroleum cokes and one coal tar pitch were used for the pilot scale anode production. Tables 1 and 2 present the properties of cokes and pitch.

**Table 1:** Physical and chemical properties of coke

Properties	Standard coke	High sulfur coke	Low sulfur coke
Bulk Density (g/cm^3^)	0.890	0.890	0.837
Real Density (g/cm^3^)	2.06	2.06	2.07
CO_2_ Reactivity (%)	9	7	8
Ash Content (%)	0.2	0.20	0.15
Moisture Content (%)	0.1	< 0.1	0.19
Na (wt%)	0.007	0.008	0.0059
Si (wt%)	0.01	0.01	0.0095
P (wt%)	0.0006	0.0006	−
S (wt%)	2.75	3.34	0.73
Ca (wt%)	0.01	0.01	0.004
V (wt%)	0.031	0.034	0.024
Fe (wt%)	0.02	0.02	0.01
Ni (wt%)	0.02	0.02	0.019

**Table 2:** Physical and chemical properties of pitch

Properties	Pitch
Ash at 900 °C (% m/m)	0.12
β Resin (% m/m)	22.2
Density at 20 °C (g/cm^3^)	1.320
Quinoline insolubles (% m/m)	6.9
Toluene insolubles (% m/m)	29.1
Coking Value (% m/m)	59.1
Softening Point (°C)	119.6
Dynamic Viscosity 170 °C (mPa.s)	1390
Surface Tension^a^ (dyne/cm) at 170 °C	39.33

### Anode production

**Fig. 1:**
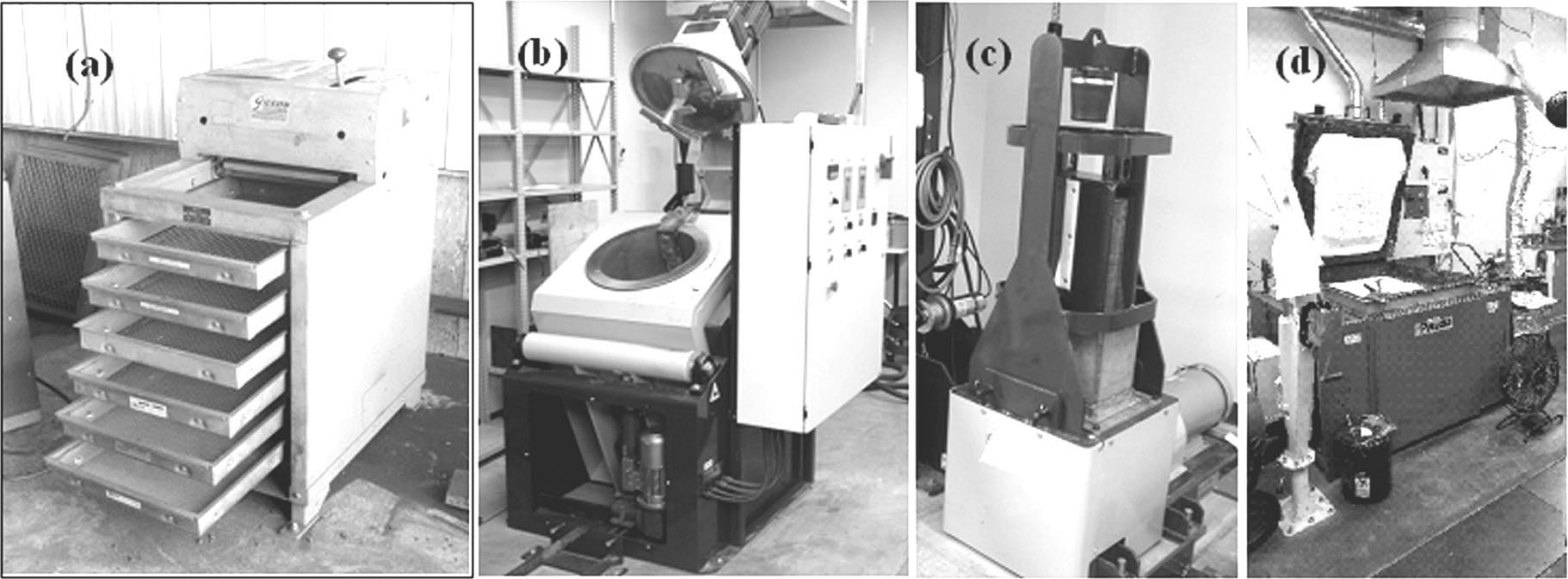
Laboratory anode production equipment: **a** sieve, **b** mixer, **c** vibro-compactor, **d** baking furnace

Anodes weighing about 10 kg were produced at the carbon laboratory of UQAC. First, the dry aggregate (coke, butt, rejected anodes) was sieved to have the desired particle size distribution required for the anode recipe (Fig. 1a). After preheating the coke and the pitch, the anode paste was prepared in an intensive mixer (Fig. 1b). The green anodes were prepared using raw materials, anode recipe, and the production conditions similar to those used in industry. Then, the paste was compacted in a vibro-compactor equipped with vacuum (Fig. 1c). The compaction was monitored with two sensors to ensure that the vertical displacement of load and the table were synchronized. At the end of compaction, the anode is removed from the mould and was allowed to cool down. The mixer and the vibro-compactor were designed by the carbon group of UQAC. The green anodes were baked in a PYRADIA (Model No-B07D02029021SVCCH) furnace (Fig. 1d). Anodes were placed in a ceramic box, covered with packing coke, and baked at a predetermined heating rate up to a desired temperature. The temperature distribution in the furnace was monitored placing a number of thermocouples inside the furnace. After baking, anodes were kept at this temperature for a certain soaking period. The properties of the laboratory anodes are similar to those of the industrial anodes [26].

#### Anode production parameters

In the first part of the study, the effect of raw materials and the vibration time on the anode quality were investigated. The quantities of ball mill product (BMP) and filter dust (FP) were kept constant. In the modified anode recipe, the percentage of coarse particles and medium particles were varied. The mixing temperature was 170 °C. The maximum baking temperature and the soaking time were 1050 °C and 8 h, respectively. The conditions were similar to the ones used by the industry except for the parameter being studied. Only the soaking time is shorter than the one used in the industry. The objective of soaking is to have a uniform temperature distribution in order to attain uniform properties in the anode since the anode properties are highly dependent on temperature. However, the industrial anodes are much bigger (around 1 ton) than the laboratory anodes. Therefore, a period of 8 h soaking time was enough for the laboratory anodes. The properties of the laboratory anodes produced were similar to those of the industrial anodes.

Table 3 summarizes the experimental conditions. Three groups of anodes were prepared and baked. In the first group of experiments, the effect of raw materials (anode recipe, butt and pitch percentage) on anode properties and cracking was studied.

**Table 3:** Experimental conditions

Anode no	FC (%), Type	Butts (%)	BR (%)	GR (%)	Pitch (%)	VT (s)	P (psi)	CM	HR (°C/h)	BT(h)
Raw material										
Anode recipe										
1*	72.5, LS	25	0.5	2	15	60	41	NCA	11	106.8
2**	72.5, LS	25	0.5	2	15	60	41	NCA	11	106.8
Butt										
3***	72.5, ST	25	0.5	2	15	60	41	NCA	11	106.8
4	62.5, ST	35	0.5	2	15	60	41	NCA	11	106.8
Pitch										
3***	72.5, ST	25	0.5	2	15	60	41	NCA	11	106.8
5	72.5, ST	25	0.5	2	13	60	41	NCA	11	106.8
6	72.5, ST	25	0.5	2	18	60	41	NCA	11	106.8
GREEN ANODE FABRICATION PARAMETERS										
Vibration time										
3***	72.5, ST	25	0.5	2	15	60	41	NCA	11	106.8
7	72.5, ST	25	0.5	2	15	72	41	NCA	11	106.8
Top-former bellow pressure										
3***	72.5, ST	25	0.5	2	15	60	41	NCA	11	106.8
8	72.5, ST	25	0.5	2	15	60	30	NCA	11	106.8
Green anode cooling medium										
3***	72.5, ST	25	0.5	2	15	60	41	NCA	11	106.8
9	72.5, ST	25	0.5	2	15	60	41	FCA	11	106.8
10	72.5, ST	25	0.5	2	15	60	41	WB	11	106.8
BAKING PARAMETERS										
Heating rate										
3***	72.5, ST	25	0.5	2	15	60	41	NCA	11	106.8
11	72.5, ST	25	0.5	2	15	60	41	NCA	15	79.2
12	72.5, ST	25	0.5	2	15	60	41	NCA	7	156.4
13	72.5, ST	25	0.5	2	15	60	41	NCA	15–7-15	108.8
14	100, ST	0	0	0	15	60	41	NCA	15	79.2
15	100, ST	0	0	0	15	60	41	NCA	11	106.8
16	100, ST	0	0	0	15	60	41	NCA	7	156.4
17	100, ST	0	0	0	15	60	41	NCA	15–7-15	108.8

*FC*, Fresh coke; BR, Baked rejects; *GR*, Green rejects; *VT*, Vibration time; *P*, Top-former bellow pressure; *CM*, Green anode cooling medium; *HR*, Heating rate; *BT*, Baking time; *LS*, Low sulfur coke; *ST* standard coke; *NCA*: Natural convection in air; *FCA*, Forced convection with air; WB: Water bath; ^*^Standard anode recipe, low sulfur coke; ^**^Modified anode recipe—low sulfur coke; ^***^Standard anode recipe—standard coke

In the second part, green anodes were made using the same raw materials but under different operating conditions (vibration time, top-former bellow pressure, and cooling medium). The effect of green anode production conditions on the anode cracking problem was studied.

In the third group, eight anodes were produced. Four of these anodes had the same percentage of butts used in a standard anode recipe and the other four anodes did not contain any butts. Anodes were baked at four different heating rates (15 °C/h, 11 °C/h, 7 °C/h, and combination (heating rate varied during baking): 15–7-15 °C/h). In this part, the effect heating rate on anode properties as well as cracking was studied in the presence and absence of butts. The evolution of crack formation at the intermediate stages of baking was given elsewhere [26, 27].

#### Anode characterization

Different properties of anodes were measured to characterize them. The measurement methods are described below.

##### Apparent density of anodes

The apparent density was determined according to the ASTM D5502-00 (2005) standard. Cylindrical cores of 50 mm diameter and 130 mm length were used. The core lengths were measured at four different positions and diameters were measured at eight positions. Their averages were used to calculate the core volume. Knowing the core weight and the volume, the density was calculated.

##### Electrical resistivity of anodes

The specific electrical resistivity was measured with anode cores of 50 mm in diameter and 130 mm in length using ASTM D6120 9 (2007).

The specific electrical resistivity distribution was measured in both green and baked anodes using a laboratory model of the SERMA (Specific Electrical Resistivity Measurement of Anodes) technology, which was developed previously by the UQAC carbon group. It consists of two plates equipped with a number of current and voltage probe pairs. When these plates come in to contact with opposing anode surfaces, a total current of 5 A is applied from the current probes on one surface. Then, the corresponding voltage drops are measured from each voltage probe pair in the vicinity of each current probe pair between the two plates using a data acquisition system. The resistivities are then calculated at different positions. The details of the equipment are given elsewhere [24, 25].

##### Anode surface crack density (external defects)

Anodes were inspected visually and the number of surface cracks were counted. The surface crack density was calculated by dividing the number of cracks by the anode surface area.

##### Tomography

Computed tomography (CT), which is an imaging technique, constructs a 3D image of an object from a numerous 2D digital images of the same object using x-ray. 2D images of vertical planes were created using CT (Somatom Sensation 64, Institute National de Recherche Scientifique (INRS-ETE)) at every 0.6 mm of the anode along the length with a resolution of 2.381 pixels per mm.

##### Optical density (tomography)

Tomography gives a distribution of optical density in the object, which is the measure of the amount of material relative to void space containing air in the object (-1000 HU (Hounsfield Unit) for air where no solid is present). Optical density values give an indication of the presence of cracks and pores, which contain air only.

##### Internal defects (tomography)

A software was developed by the UQAC carbon group using Matlab R2015a to analyze the hundreds of 2D images (on vertical planes along the height) of green and baked anodes and estimate the percentage of internal defects in the anode. Information obtained from all 2D images in a given anode permits the quantification of the results within its volume. The results are presented as one image per anode showing the total internal defect distribution for that anode.

## Results and discussion

### Tomographic images and electrical resistivity measurements

**Fig. 2:**
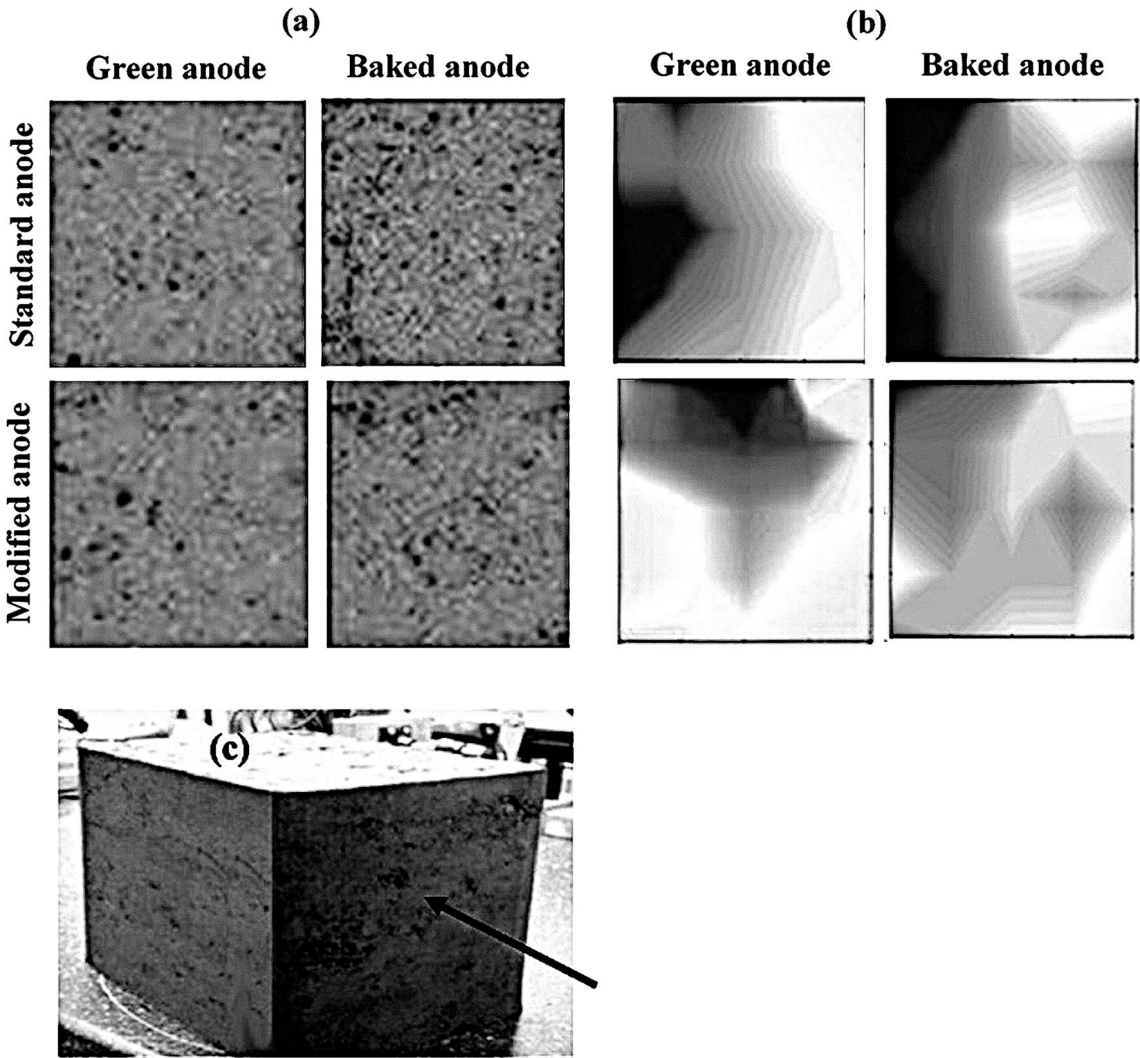
**a**: Map of internal defects determined from the 2D tomography images and **b** Electrical resistivity maps of green and baked anodes between the small side surfaces (along the length) of standard and modified anodes; **c** Small side surface of the laboratory anode

The total number of defects in anodes was determined with the software developed by the UQAC carbon group to analyze the numerous 2D images taken during tomography. An example is given in Fig. 2a for anodes 3 and 4 (Table 3). Images showing all defects along the length are presented in Fig. 2a for both green and baked anodes produced with different butt contents. Darker regions correspond to higher number of internal defects. The resistivity maps between the small side surfaces of anodes, which were measured with the equipment developed at UQAC (SERMA), were also determined [24, 25]. These for the same cases are shown in Fig. 2 (b). Darker regions have higher resistivities. Figure 2c shows a lab anode and the direction of measurement. It should be noted that the green anode electrical resistivities are about two orders of magnitude greater (above 1500 μΩ·m) than those of the baked anodes. Small variations in pitch concentration in green anodes could give significant differences in the resistivity values. The baked anode resistivities, on the other hand, directly indicate the presence of pores and cracks. These figures show that the internal number of defects is higher for both green and baked anodes containing greater butt percentage compared to the standard anode. The electrical resistivities also show a similar trend. From these figures, the agreement between the tomography and SERMA results can be seen: the zones that contain more defects (determined by the tomography) have higher resistivities (determined by SERMA). Also, the comparison of the baked and green anode resistivities shows that generally a high resistivity zone in a green anode results in a high resistivity zone after baking.

These analyses were carried out for all the cases in Table 3. Then, the overall results are determined and presented in the following section.

### Effect of raw materials

In this part, the effects of anode recipe and butt and pitch contents on the cracking and anode properties were investigated (Table 3).

#### Anode recipe

Two anodes (anodes 1 and 2, Table 3) were produced using different particle size distributions. In anode 1, a standard anode recipe was used whereas the anode 2 was produced with a modified recipe. The recipe modification and its effect on anode properties were previously studied [2]. In this study, its effect on crack (defect) formation is studied. In the modified recipe, the fractions of medium and coarse particles were readjusted. The ultra-fine fractions (ball mill product (BMP) and filter dust (FD)) were kept similar to that of the standard recipe.

**Fig. 3:**
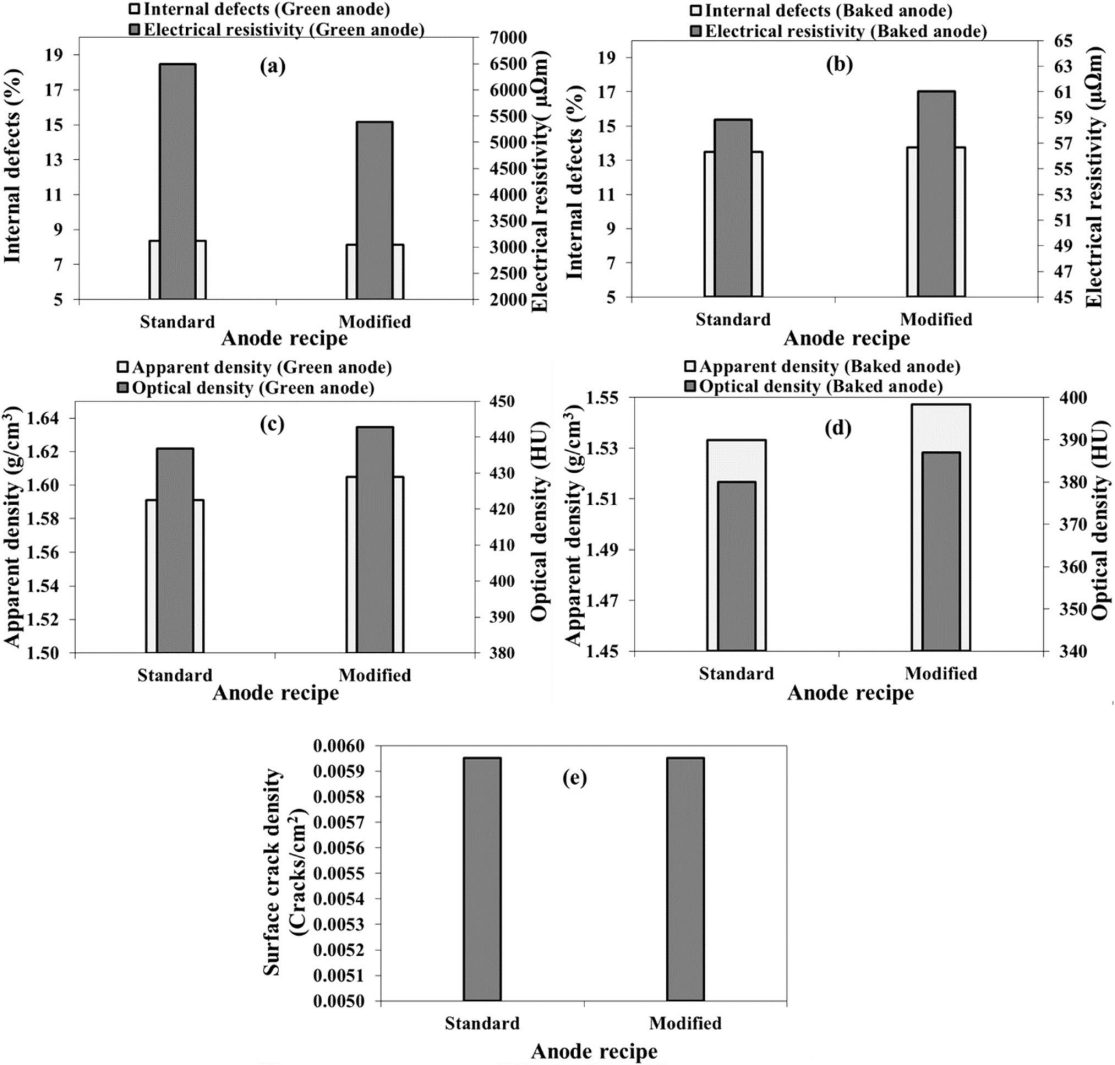
Effect of anode recipe on **a–b**: internal defect percentage and electrical resistivity, **c–d** apparent and optical densities, and **e** surface crack density in **a, c** green and **b**, **d**, **e** baked anodes

Comparing the electrical resistivity of the two green anodes (Fig. 3a) shows that the resistivity decreased when the recipe is modified; however, the number of defects remained practically the same. This indicates that the difference in resistivity values is due to slight differences in pitch distribution. On the other hand, after baking (Fig. 3b), the anode made with the modified recipe is found to have a higher resistivity compared to the anode prepared with the standard recipe. This shows that the modified recipe is likely to cause the creation of somewhat more cracks during baking. The internal defects of the baked anode made using modified recipe is slightly higher compared to the standard anode.

A good anode density is generally considered as a sign of a good quality anode. Figure 3c presents the apparent and optical density of two green anodes made with different recipes and Fig. 3d presents the densities of the same anodes after baking. It can be seen that the apparent densities of both the green and the baked anodes were slightly improved when the anode recipe was modified. The optical densities of the anodes also show the same trend. The optical density, which is obtained from the tomographic analysis, indicates the relationship between solid and void space in materials. Higher optical density was obtained for the modified aggregate which means that the solid content is higher than the void space in this anode compared to the standard anode both before and after baking. The results of the tomographic analysis results agree with the direct measurements of the densities. The surface crack densities of both anodes are similar (Fig. 3e). The surface cracks are not good indicators of anode quality since an anode with surface cracks can have an interior structure of acceptable quality.

#### Butt content

**Fig. 4:**
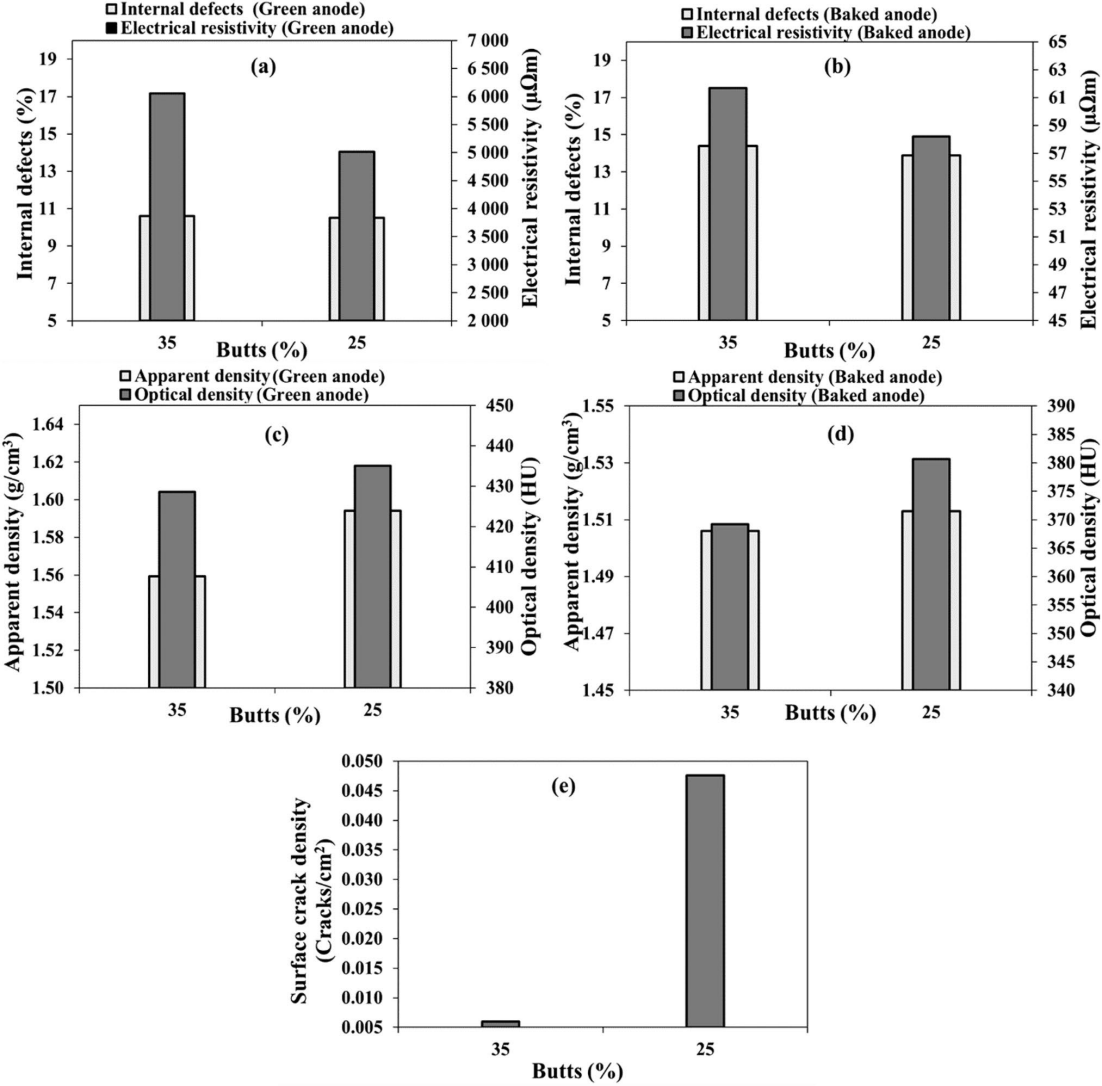
Effect of butt content on: **a, b** internal defect percentage and electrical resistivity, **c, d** apparent and optical densities, and **e** surface crack density in **a**, **c** green and **b**, **d**, **e** baked anodes

Anodes 3 and 4 have different butt contents (25% and 35%, Table 3). Figure 4a and b show that increasing butt content did not influence the internal defect percentage in green anodes but increased it slightly in baked anodes. The electrical resistivity increased as the butt content increased both for green and baked anodes. The impact of higher butt content may be explained with the low wettability of butt with pitch matrix [28] and the difference in thermal expansion coefficients of coke and butt [2–4, 26]. This difference might create stress, consequently, anodes might crack during baking. It might also be due to the insufficient pitch penetration into the butt pores. The results also show that increasing butt content decreased the anode apparent and optical densities both for green and baked anodes (Fig. 4c and d). It should be noted that the percentage of pitch was not adjusted when the percentage of butt was changed. In general, the effect of butts on anode properties depends on the butt quality. Visual inspection of the baked anodes indicated that the surface cracks decreased when the butt content was increased (Fig. 4e). As it was mentioned previously, surface cracks do not necessarily represent the internal anode quality.

#### Pitch content

**Fig. 5:**
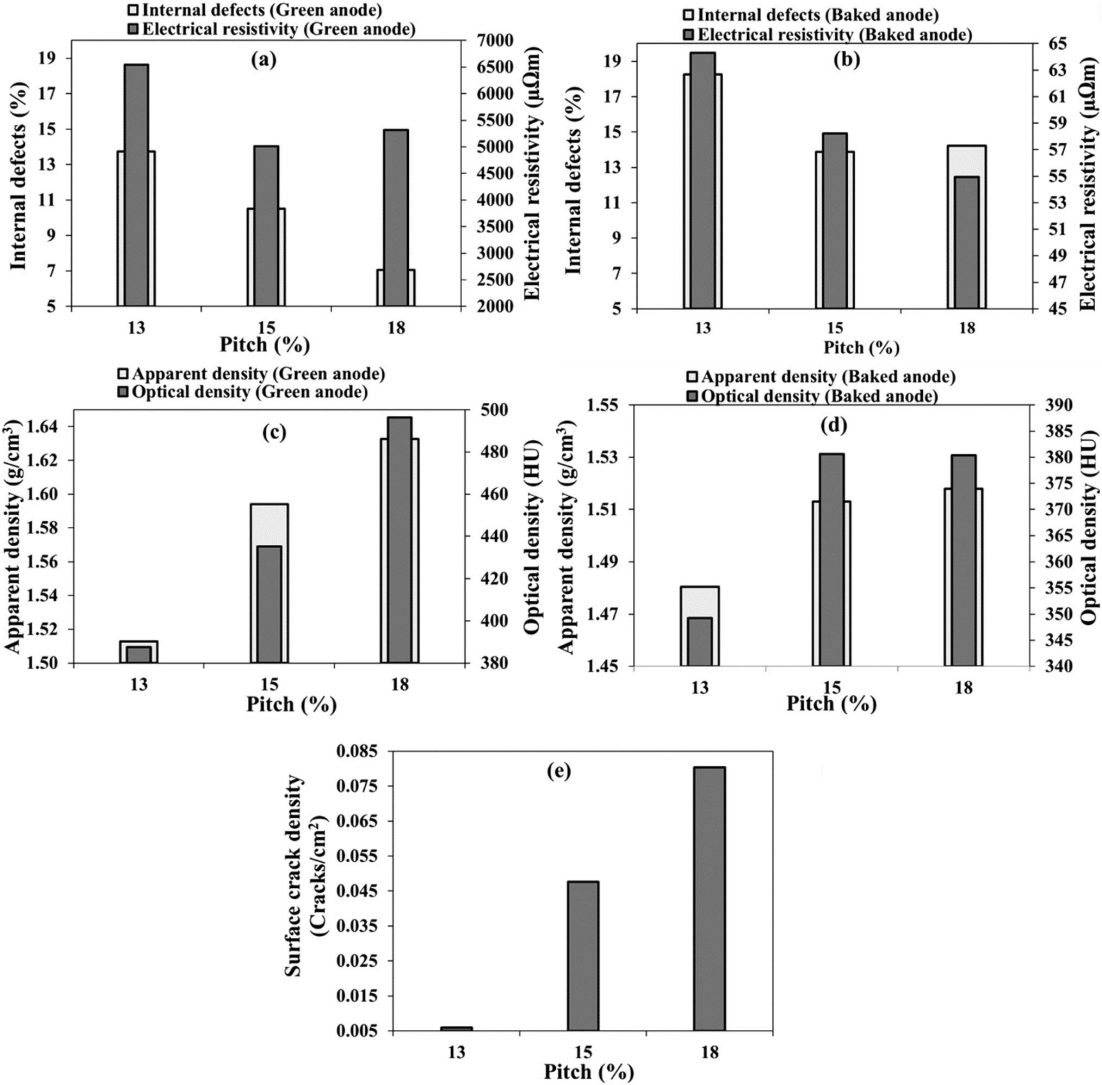
Effect of pitch content on: **a–b** internal defect percentage and electrical resistivity, **c–d** apparent and optical densities, and **e** surface crack density in **a**, **c** green and **b**, **d**, **e** baked anodes.

Three different pitch percentages (13%, 15%, and 18%) were used to study the effect of this parameter on the quality of the anodes (anodes 3, 5, and 6, Table 3). The results show that the increase in the percentage of pitch decreases the internal defect percentage for green anodes (Fig. 5a) as expected. When there is not enough pitch, pores of coke and the interparticle spaces are not completely filled, resulting in a porous anode [29].

Electrical resistivity of green anodes decreased with increasing pitch content up to a certain pitch percentage (15%), which was close to optimum pitch content. However, the electrical resistivity increased with further increase in pitch percent. When pitch fills the voids between the coke particles and the pores in the particles of green anodes, the electrical resistivity decreases. Nevertheless, if the pitch percentage is increased more, the accumulation of excess pitch between the particles increases the electrical resistivity [3, 29]. This can be seen in Fig. 5a where the anode produced using 15% pitch had lower electrical resistivity than the other two anodes containing 13% and 18% pitch. For baked anodes, increasing the percentage of pitch decreased the resistivity as shown in Fig. 5b. Internal defects were slightly higher for the baked anode containing the highest pitch percentage, which was over-pitched to some extent. Over-pitching can cause crack formation during baking due to the higher quantity of released volatiles (Fig. 5b).

As it can be seen in Fig. 5c, both the optical and apparent densities increased with increasing pitch percent for green anodes. The baked anode optical and apparent densities increased appreciably when the pitch content is increased from 13 to 15%. However, further increase in pitch percent did not have a significant effect on the densities (Fig. 5d). The apparent density increased slightly whereas the optical density remained the same.

The results of the visual inspection (surface cracks from Fig. 5e) show that increasing the amount of pitch can cause many surface cracks, but this does not reveal the internal quality. The anode with a high percentage of pitch has a low electrical resistivity and low internal defects compared to those of the anode made with 13% pitch (Fig. 5b), but it has high surface crack density (Fig. 5e). This again shows that the quality of anodes cannot be evaluated correctly only with the visual inspection of the surface.

### Green anode fabrication parameters

#### Vibration time

**Fig. 6:**
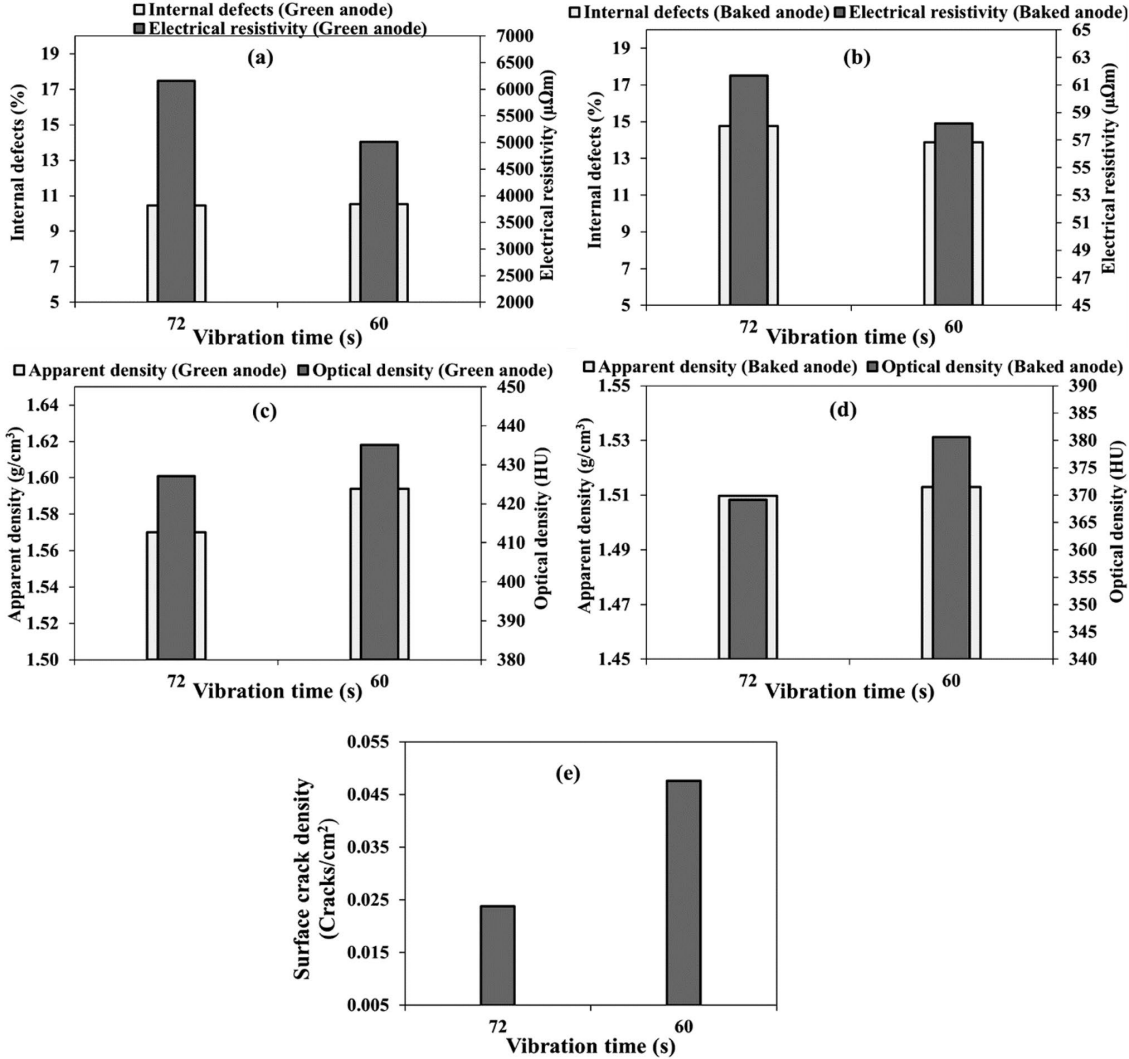
Effect of vibration time on: **a–b** internal defect percentage and electrical resistivity, **c–d** apparent and optical densities, and **e** surface crack density in **a, c** green and **b**, **d**, **e** baked anodes

Two anodes (anodes 3 and 7) were produced using vibration times of 60 and 72 s (Table 3). Figure 6 shows the effect of vibration time on internal defects, electrical resistivity, apparent and optical densities, and specific surface crack density.

The green and baked anodes produced using lower vibration time has lower resistivities than those of the anodes produced using a higher vibration time. This result indicates that the anode was over-compacted when 72 s was used. Over-compaction (too high a vibration time) causes more stress accumulation in the green anode. Both green anodes have similar amounts of defects. However, baked anode produced using a lower vibration time has a slightly lower defect percentage. Accumulation of stress during green anode formation results in defect formation during baking. The electrical resistivity is lower for the anode produced using a lower vibration time for both green and baked anodes (Fig. 6a and b). However, there is a limit for decreasing the vibration time. If the anodes are not compacted enough (under-compaction), this also increases the internal defects and the resistivity. It is important to find the optimum vibration time necessary for a given anode [16].

The results show that both green and baked anodes produced using the low vibration time has a higher apparent and optical densities than those of the anodes made with the higher compaction time (Fig. 6c and d).

The anode manufactured using a low vibration time has more surface cracks than that manufactured with a high compaction time (Fig. 6e). Visual inspection does not indicate the internal anode quality. The internal defect analysis show that the quality of this anode is better than the over-compacted anode (Fig. 6a and b) even if it has more surface cracks.

#### Top-former bellow pressure

**Fig. 7:**
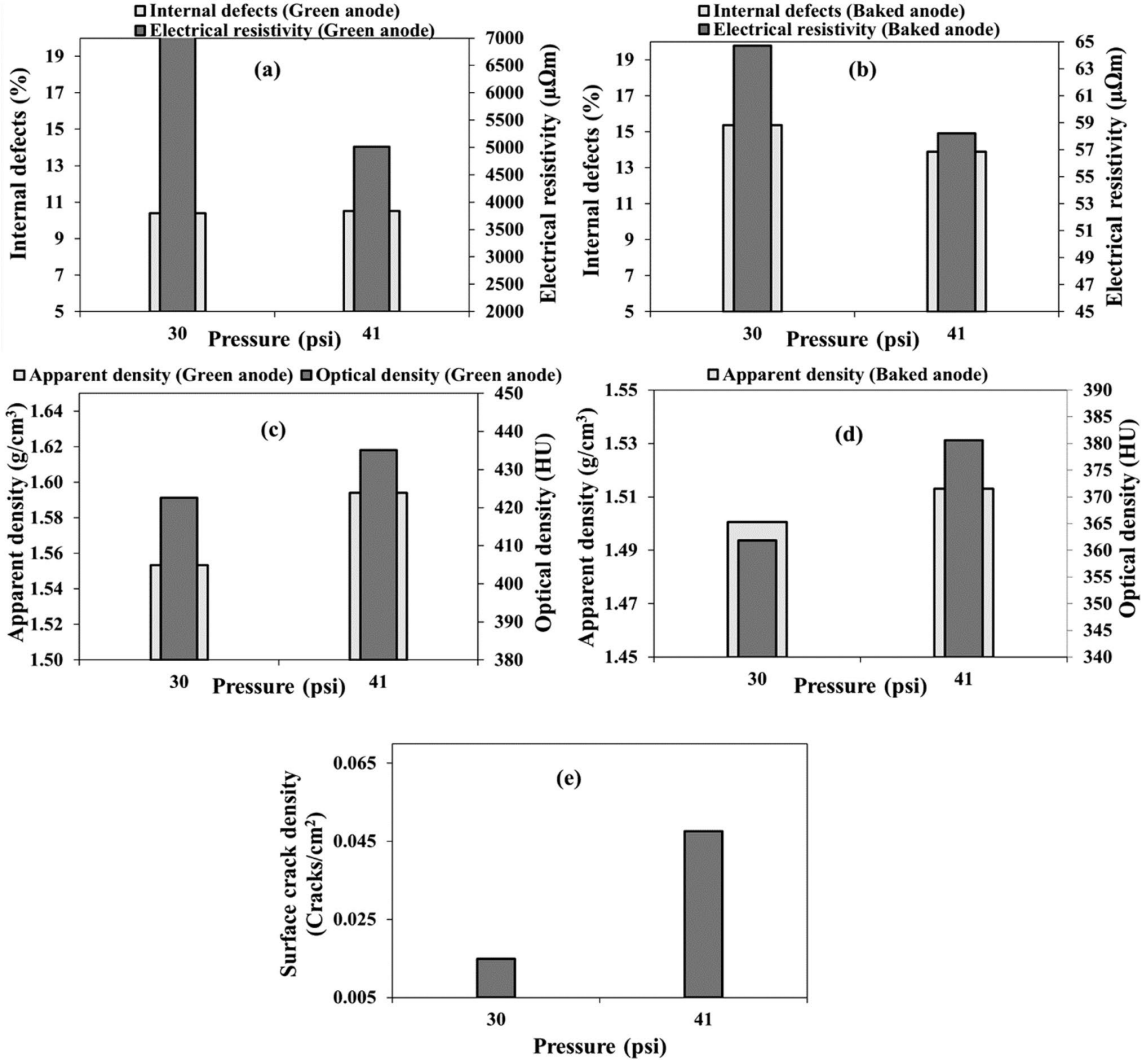
Effect of top-former bellow pressure on: **a–b** internal defect percentage and electrical resistivity, **c–d** apparent and optical densities, and **e** surface crack density in **a**, **c** green and **b**, **d**, **e** baked anodes

Two anodes (anodes 3 and 8) were made using a top-former bellow pressure of 41 and 30 psi (Table 3). The electrical resistivity decreased as the pressure increased both for green and baked anodes (Fig. 7a and b). Under-compaction (in this case, due to an insufficient pressure) led to poor anode quality. Less pitch penetrated between the particles and in the particle pores, increasing the electrical resistivity. The percentage of internal defects for the two green anodes were similar (Fig. 7a) whereas those of the baked anodes were clearly different. The baked anode manufactured with the low compaction pressure had more internal defects than that manufactured using a higher pressure. Under-compaction makes the matrix weak and facilitates the formation of pores and cracks (Fig. 7b). If the anode has more defects, its electrical resistivity also increases as pores and cracks form a barrier to the passage of an electric current (discontinuous solid medium).

As expected, the anode manufactured under a low compaction pressure has a lower density (both apparent and optical) than the anode manufactured at the higher compaction pressure (Fig. 7c and d). The baked anode compacted at higher pressure had more surface cracks which did not again reflect the overall quality of the anode (Fig. 7e).

### Green anode cooling medium

**Fig. 8:**
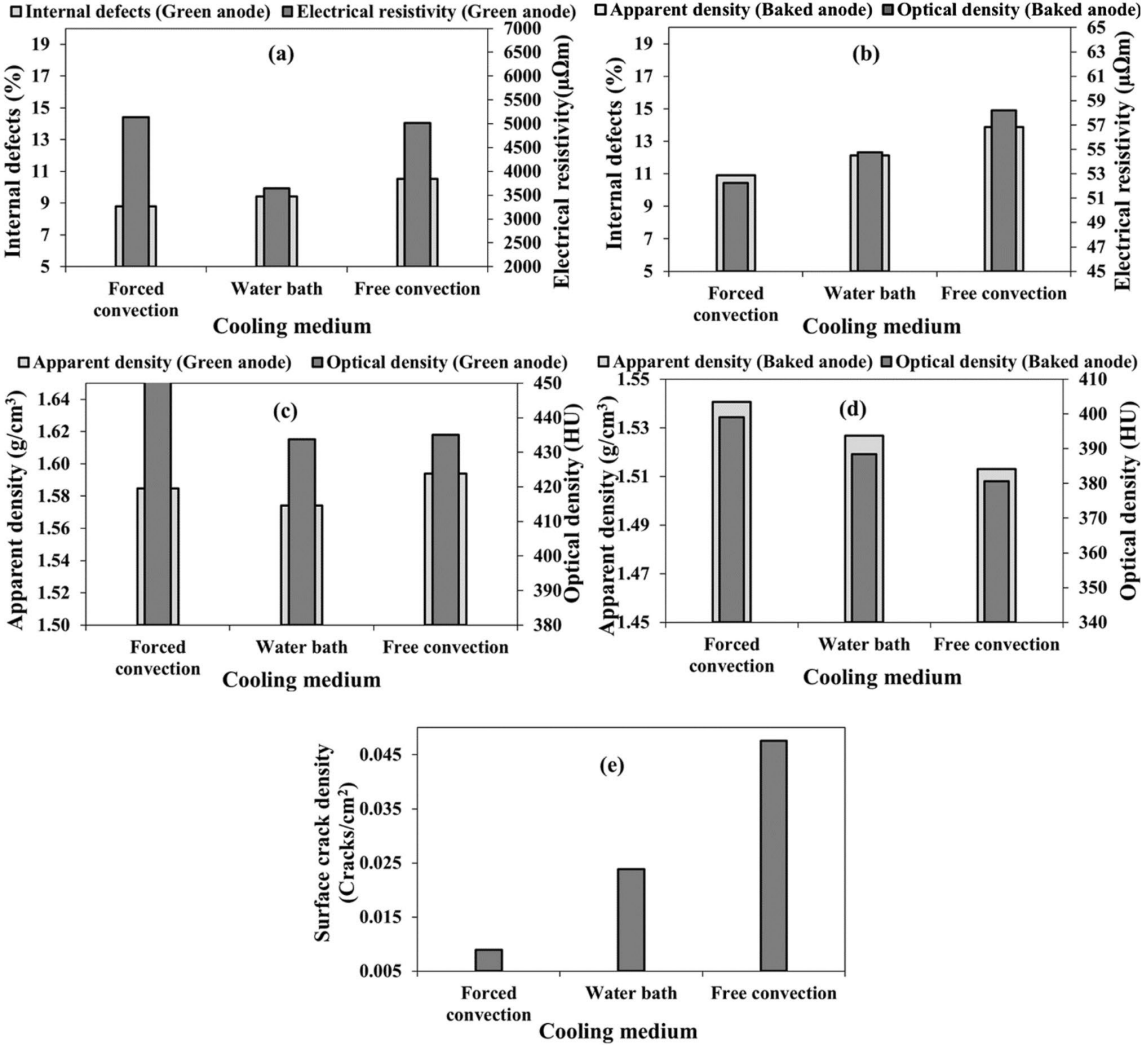
Effect of cooling medium on: **a–b** internal defect percentage and electrical resistivity, **c–d** apparent and optical densities, and **e** surface crack density in **a**, **c** green and **b**, **d**, **e** baked anodes

Three green anodes (anodes 3, 9 and 10, Table 3), which were produced under the same conditions, were cooled differently: free convection in air, forced convection in air, and forced convection in a water bath. Cooling was stopped when the measured temperature reached the temperature of the cooling medium. The percentage of internal defects was the lowest for the anode cooled by forced convection in air and the highest for the one cooled by free convection in air both for green and baked anodes. The electrical resistivity was lowest when the anode was cooled by immersing in water bath for green anodes, and the resistivities of the green anodes cooled with forced and free convection in air were similar. The resistivity of a green anode is generally influenced by pores/defects as well as by pitch distribution. Non-carbonized pitch has a high electrical resistivity. If there are high pitch regions, an increase in resistivity occurs in those regions [3, 26]. Also, the cooling rate is lower in air, which leads to a more porous anode due to the spring-back effect. For baked anodes, the resistivity and the number of internal defects were the lowest for the anode cooled by forced convection in air and the highest for the one cooled by free convection in air (Fig. 8a and b). In addition, the apparent and optical densities of baked anodes, which were cooled using forced convection in air after green anode is formed, were the highest. For the green anode, the apparent density was highest for the anode cooled with free convection in air whereas the optical density was highest for the anode cooled with forced convection in air. The presence of impurities in the raw material could also affect the optical density (Fig. 8c and d). The surface cracks were also lowest for the anode cooled with forced convection in air (Fig. 8e). In general, the results indicate that the forced convection in air seems to be the best option for cooling green anodes among the three options tested in this study. It must be noted that there are other parameters that need to be studied such as water and air temperatures, air flow rate for the forced convection, combination of water/air cooling media, etc. before the most suitable cooling medium could be selected.

### Heating rate used during baking

Eight anodes, four without butts (anodes 14, 15, 16, and 17) and four containing butts (anodes 3, 11, 12, and 13), were baked using low (7 °C/h), medium (11 °C/h), high (15 °C/h), and combination (15–7–15 °C/h) heating rates (Table 3). Concerning the combination heating rate, after initially using a high heating rate at low temperatures, a low heating rate was used during the pitch devolatilization period since most of the cracks form during this period. The rest of the baking was carried out at the highest heating rate. Baking time depends on the heating rate used. Usually, the lowest heating rate is the best since the volatiles are released slowly during pitch carbonization, and this prevents the formation of high internal pressure in the anode during volatile release and consequently reduces the cracks formation [30]. However, this increases the anode production time and cost, and decreases the production rate. The combination heating rate scheme was proposed since the baking during the critical period was carried out at a low heating rate without increasing the total production time compared to that of standard heating rate (medium) (Table 3).

#### Anodes without butts

**Fig. 9:**
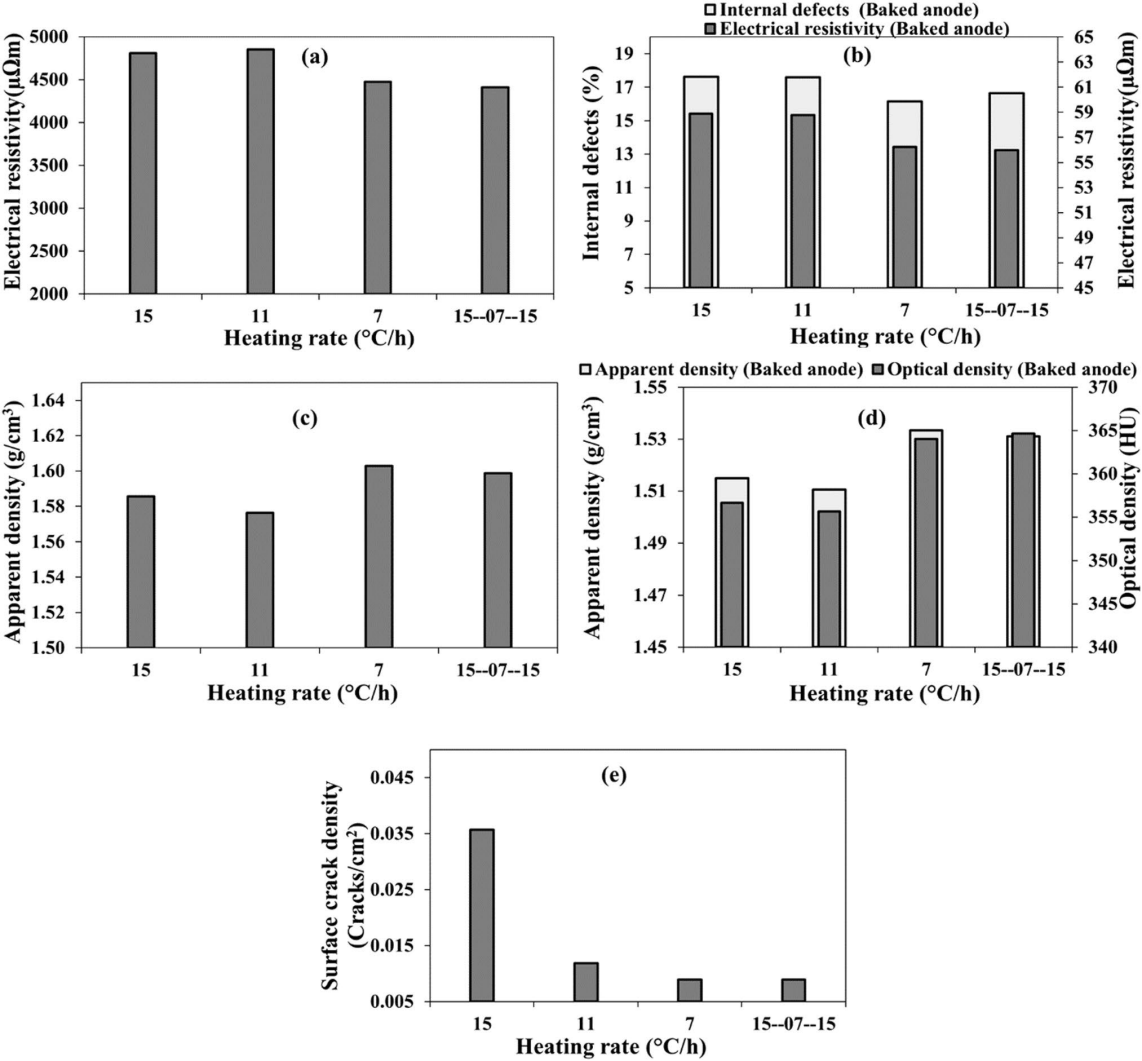
Effect of heating rate on: **a** electrical resistivity, **b** internal defect percentage and electrical resistivity, **c** apparent density, **d** apparent and optical densities, and **e** surface crack density in **a**, **c** green anodes baked later with shown heating rates and **b**, **d**, **e** baked anodes (without butts)

The electrical resistivities and apparent densities of the anodes before baking (green anodes) are given in Fig. 9a and c. As it can be seen from these figures, there are some differences in these properties of the green anodes although they are produced under the same conditions. This is expected since the anode raw materials are non-homogeneous, resulting in small differences in properties. The effect of heating rate on the internal cracks and electrical resistivity are presented in Fig. 9b whereas their effect on the apparent and optical densities are given in Fig. 9d for baked anodes. Figure 9e presents the surface crack density of the baked anodes.

The results show that the internal defect percentage and electrical resistivity of anodes baked at the lowest and combination heating rates are similar and lower than those of the anodes baked using medium and high heating rates. In addition, the anodes baked at the lowest and combination heating rates have higher apparent and optical densities than the other two anodes baked at medium and high heating rates. The surface crack density also decreased with decreasing heating rate and the anode baked at the combination heating rate had a similar surface crack density to the anode baked at the lowest heating rate (Fig. 9e). It seems that the better-quality anodes are produced using the lowest and combination heating rates; however, it must be noted that their corresponding green anodes had better quality before baking. Therefore, more testing is needed to confirm the positive impact of low heating rate on anode quality.

#### Anodes with butts

**Fig. 10:**
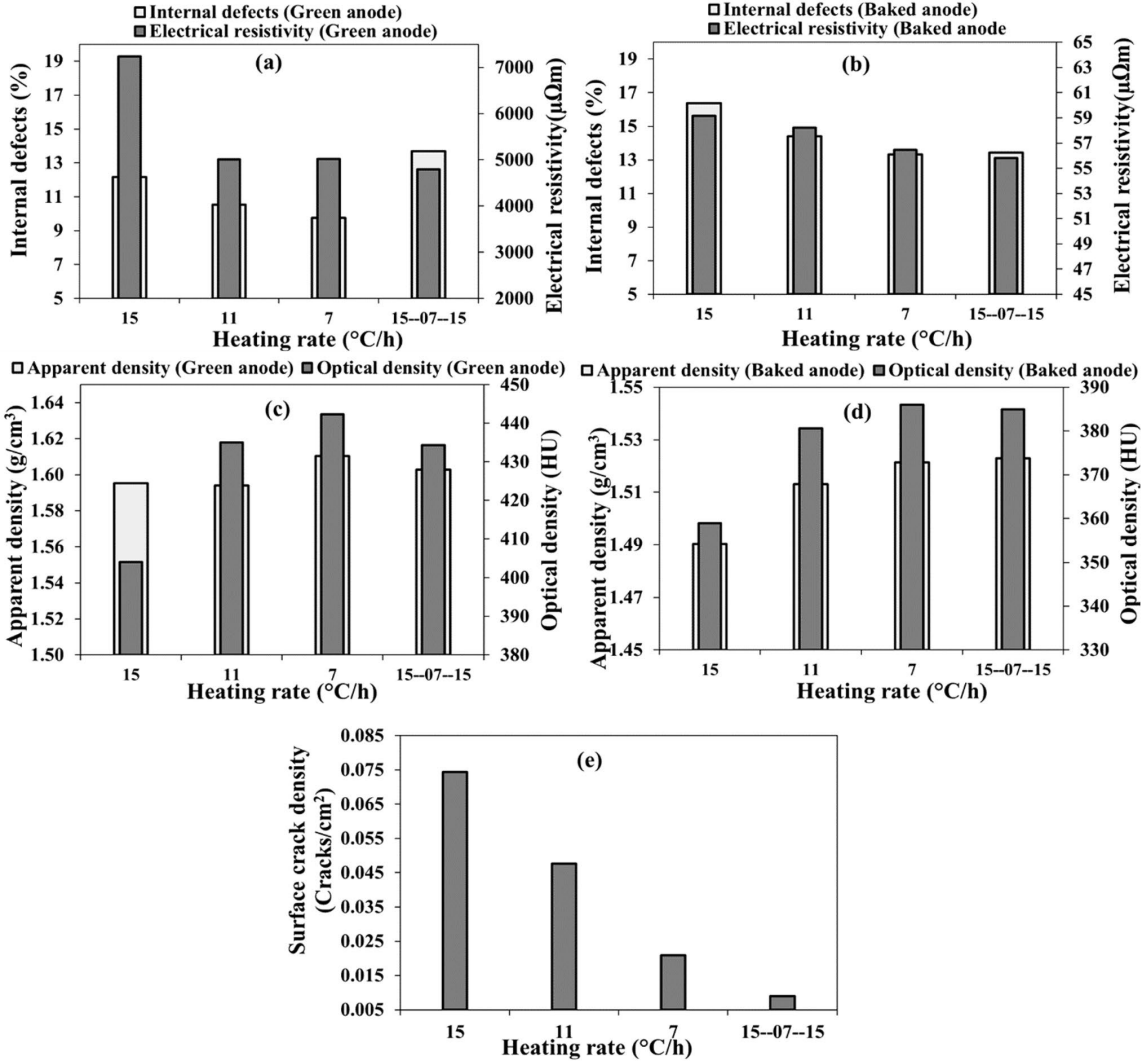
Effect of heating rate on: **a** electrical resistivity, **b** internal defect percentage and electrical resistivity, **c** apparent density, **d** apparent and optical densities, and **e** surface crack density in **a**, **c** green anodes baked later with shown heating rates and **b**, **d**, **e** baked anodes (with butts)

Four other anodes, this time with the butt addition, were produced and baked using the four heating rates explained in the previous section. The properties of green anodes used are given in Fig. 10 a and c. The heating rates shown in these figures indicate the heating rate used when these green anodes were baked. It is difficult to make green anodes with the same properties due to the non-homogeneity of the raw materials. Although, the green anode, which was later baked using the combination heating rate, had the highest internal crack before baking, the corresponding baked anode had the lowest internal cracks among the anodes tested (Fig. 10b). In addition, this anode also had high anode apparent and optical densities after baking even if it had lower green anode densities compared to that of the green anode baked at the lowest heating rate and similar to that of the green anode baked at the medium heating rate (Fig. 10b). The anode baked with the combination heating rate also had the lowest resistivity. As it can be seen from the Fig. 10a and b, although the anode baked at the medium heating rate had similar electrical resistivity to those baked using the low and combination heating rates in green state, it had higher resistivity after baking when compared to those of the same anodes in baked state. The properties of the anode baked using the combination heating rate were similar to those baked at the lowest heating rate. Its surface crack density was also the lowest (Fig. 10e). After, these tests, it now possible to state that the lowest and combination heating rates give better quality anodes. The combination heating rate has the advantages of shorter production time (similar to that baked at medium heating rate, which is the average heating rate usually observed in industry) compared to the anode produced using the low heating rate.

### Correlation between anode properties and anode quality

**Fig. 11:**
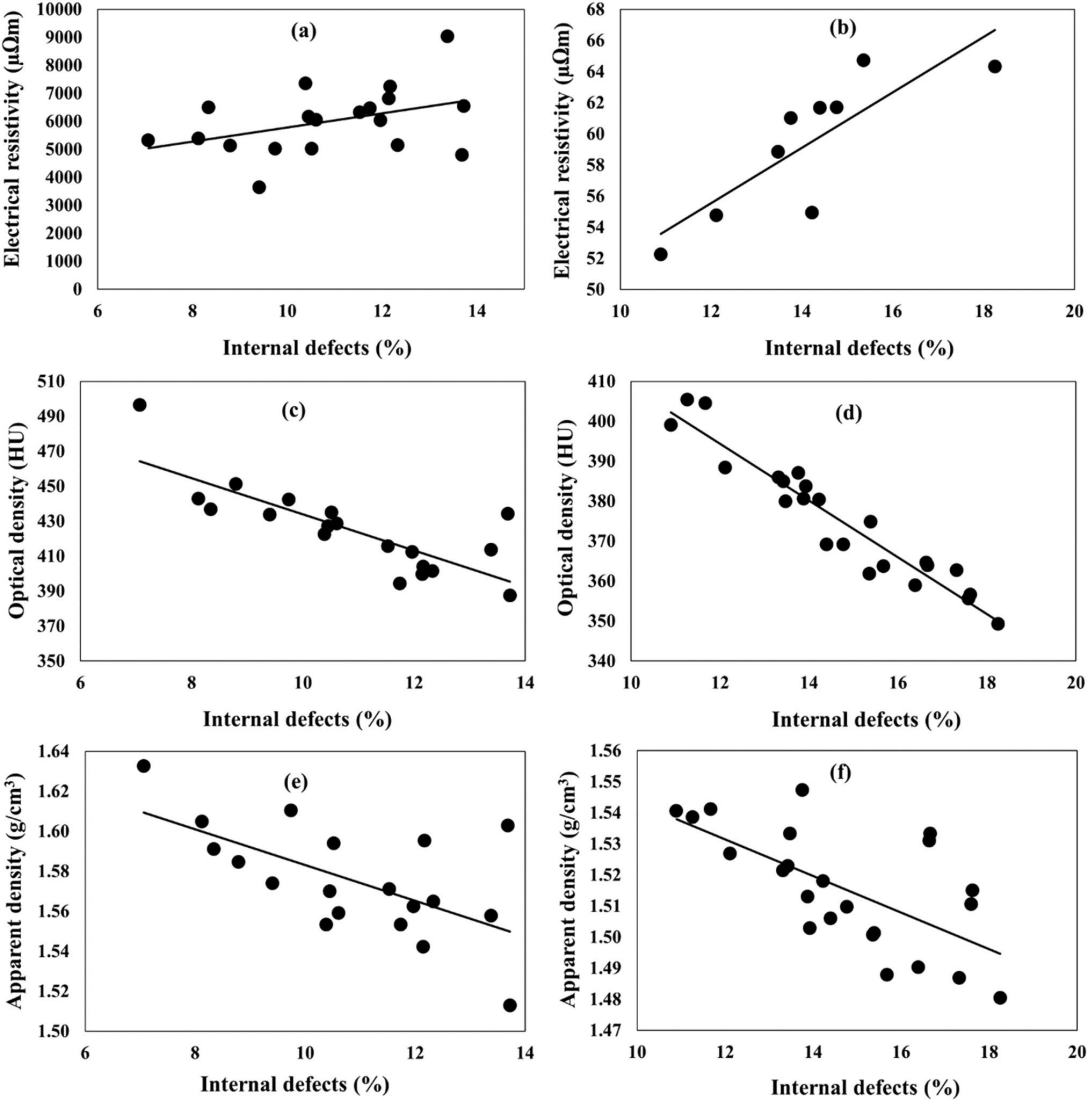
Correlation between **a–b** electrical resistivity, **c–d** optical density, and **e**–**f** apparent density and internal defect percentage: electrical resistivity for **a**, **c**, **e** green and **b**, **d**, **f** baked anodes

The Fig. 11a and b show the effect of internal defect (cracks/pores) percentage on the electrical resistivity for green and baked anodes, respectively. Electrical resistivity increased as the internal defect percentage increased for both anodes. This means that the anode quality decreases with increasing internal defects since higher resistivity indicates higher energy consumption to produce the same quantity of aluminum. The high resistivity indicates the presence of pore/cracks as well as the local high pitch regions in green anodes since pitch has high resistivity before carbonization (baking); and the high resistivity is due to defects/pores in baked anodes.

Similarly, it can be seen from the optical and apparent density vs. internal defect percentage data that the anode density, hence the anode quality, decreased with increasing internal defects both for green (Fig. 11c and d) and baked anodes (Fig. 11e and f). This is in agreement with the resistivity data. These results show the tendencies expected with respect to the relation between various parameters.

## Conclusions

The quality of anode is related to the internal defects since anode properties are affected by the presence of pores and cracks. In this study, the effect of different anode production parameters on anode properties (electrical resistivity, optical and apparent densities) were studied. Increasing pore/crack percentage decreases anode density and increases electrical resistivity. Lower anode density results in shorter anode life (lower production and higher gas emissions per unit carbon used) whereas lower anode resistivity indicates greater power consumption and higher cost per unit aluminum produced. Therefore, it is crucial to know where on the production line these defects are formed and take the necessary precautions to prevent their formation as much as possible.

The results showed that raw materials significantly affect the anode quality. Using high butt percentage increases the defects. It is important to use enough pitch. Over and under-pitching the anodes increase the internal defects. Process parameters such as vibro-compaction time, the top-former bellow pressure used during compaction, and the type of media used to cool the green anodes were studied. The compaction parameters should be as close to their optimum values as possible since over and under-compaction of green anodes result in defect formation. Cooling anodes with forced convection in air gave the best quality green anodes under the conditions studied.

Baking is one of the most important steps in anode production which plays an important role in defining the final anode quality. If the baking conditions are not chosen well, even the best quality green anode might result in a poor quality-baked anode. One of the important baking parameters is the heating rate. This study showed that the anode quality is better if the anodes are baked at a low heating rate. However, in practice, the utilization of low heating rate reduces the production and increases the cost. Most of the cracks form during the devolatilization period while the pitch is carbonizing. If the heating rate is high, high quantity of volatiles are released in a short time interval. This increases the pressure inside the anode causing cracking. When the heating rate is low, the volatiles are release slowly over a longer period of time. In this case, the crack formation is reduced due to lower internal pressure build-up inside the anode. Therefore, a combination heating rate was proposed to produce good quality anodes without increasing the production rate and cost. In combination heating rate, it is recommended to use a low heating rate during the devolatilization period and high heating rate at other times. The application of such a heating rate variation as a function of the temperature range in the plant will require the reconsideration of the furnace control and testing on site.
